# *Arachis hypogaea* gene expression atlas for *fastigiata* subspecies of cultivated groundnut to accelerate functional and translational genomics applications

**DOI:** 10.1111/pbi.13374

**Published:** 2020-04-23

**Authors:** Pallavi Sinha, Prasad Bajaj, Lekha T. Pazhamala, Spurthi N. Nayak, Manish K. Pandey, Annapurna Chitikineni, Dongxin Huai, Aamir W. Khan, Aarthi Desai, Huifang Jiang, Weijian Zhuang, Baozhu Guo, Boshou Liao, Rajeev K. Varshney

**Affiliations:** ^1^ Center of Excellence in Genomics and Systems Biology (CEGSB) International Crops Research Institute for the Semi‐Arid Tropics (ICRISAT) Hyderabad India; ^2^ Department of Biotechnology University of Agricultural Sciences (UAS) Dharwad India; ^3^ Oil Crop Research Institute (OCRI) Chinese Academy of Agricultural Science (CAAS) Wuhan China; ^4^ College of Plant Protection Fujian Agriculture and Forestry University (FAFU) Fuzhou China; ^5^ USDA‐ARS Crop Protection and Management Research Unit (CPMRU) Tifton GA USA

**Keywords:** *Arachis hypogaea*, allergens, gene expression atlas, gravitropism, oil biosynthesis, photomorphogenesis

## Abstract

Spatio‐temporal and developmental stage‐specific transcriptome analysis plays a crucial role in systems biology‐based improvement of any species. In this context, we report here the *Arachis hypogaea* gene expression atlas (AhGEA) for the world's widest cultivated subsp. *fastigiata* based on RNA‐seq data using 20 diverse tissues across five key developmental stages. Approximately 480 million paired‐end filtered reads were generated followed by identification of 81 901 transcripts from an early‐maturing, high‐yielding, drought‐tolerant groundnut variety, ICGV 91114. Further, 57 344 genome‐wide transcripts were identified with ≥1 FPKM across different tissues and stages. Our in‐depth analysis of the global transcriptome sheds light into complex regulatory networks namely gravitropism and photomorphogenesis, seed development, allergens and oil biosynthesis in groundnut. Importantly, interesting insights into molecular basis of seed development and nodulation have immense potential for translational genomics research. We have also identified a set of stable expressing transcripts across the selected tissues, which could be utilized as internal controls in groundnut functional genomics studies. The AhGEA revealed potential transcripts associated with allergens, which upon appropriate validation could be deployed in the coming years to develop consumer‐friendly groundnut varieties. Taken together, the AhGEA touches upon various important and key features of cultivated groundnut and provides a reference for further functional, comparative and translational genomics research for various economically important traits.

## Introduction

Cultivated groundnut or peanut (*Arachis hypogaea* L), one of the leading legume and oilseed crop with high oil content, nutritional and protein values, plays a significant role in ensuring nutritional food security in many developing countries in Asia and Africa (Varshney *et al.*, 2013). Due to its multiple use in the form of seed, fodder, processed oil and cake, groundnut contributes to improving the livelihood of the smallholder farmers. This crop faces an array of biotic and abiotic stress challenges in addition to certain food safety issues caused by aflatoxin and allergens to consumers' health (Pandey *et al.*, 2019a). It is essential to have an immaculate understanding of key agronomic and stresses including climate change impact on groundnut productivity for breeding better varieties.

Cultivated tetraploid groundnut being host to two different subgenomes (A and B) poses several challenges in conducting structural and functional genomics studies (Pandey *et al.*, 2016). Nevertheless, availability of reference genomes for diploid progenitor species, that is *A. duranensis* (Bertioli *et al.*, 2016; Chen *et al.*, 2016a) and *A. ipaensis* (Bertioli *et al.*, 2016), has accelerated the pace of genomics research in groundnut. Additionally, recently available reference genomes for both the subspecies of cultivated tetraploid groundnut (Bertioli *et al.*, 2019; Chen *et al.*, 2019; Zhuang *et al.*, 2019) are likely to enhance the precision in genomics research. However, mere availability of genome sequences is not enough for developing better understanding on key traits, and a holistic ‘Omics approach’ or systems biology research is required wherein the information from genomics, transcriptomics, proteomics, metabolomics, epigenomics and interactomics studies is integrated. In this context, development of a global genome‐wide gene expression atlas is essential to understand the flow of genetic information in addition to trait‐ and tissue‐specific transcriptomes.

Gene expression atlases have been developed for several plant species, including *Arabidopsis thaliana* (model plant; Schmid *et al.*, 2005), *Medicago truncaluta* (model crop for legumes; Benedito *et al.*, 2008), *Oryza sativa* (model crop for cereals; Wang *et al.*, 2010), *Glycine max* (model crop for oilseeds; Severin *et al.*, 2010), *Zea mays* (Sekhon *et al.*, 2011), *Pisum sativum* (Alves‐Carvalho *et al.*, 2015), *Vigna unguiculata* (Yao *et al.*, 2016), *Cajanus cajan* (Pazhamala *et al.*, 2017) and *Cicer arietinum* (Kudapa *et al.*, 2018). These studies aimed to investigate the complex and distinct biological processes involved in pod development, seed formation, seed maturation and oil biosynthesis. Availability of global transcriptome technologies offered the opportunity to understand the cascade of a transcriptional pattern of plant growth and development (Sekhon *et al.*, 2011). For example, the developmental process must change during the course of evolution with different morphological forms and altered plant structure.

Of the two subspecies (*fastigiata* and *hypogaea*) of cultivated groundnut, the subsp. *fastigiata* covers highest groundnut cultivated area in the world, mostly in Asia and Africa. In this context, similar to the reference genomes developed for subsp. *hypogaea* (Bertioli *et al.*, 2019) and subsp. *fastigiata* (Chen *et al.*, 2019; Zhuang *et al.*, 2019), it is necessary to develop global gene expression atlas for both the subspecies. In fact, a transcriptome map has been developed for subsp. *hypogaea*, mostly limited to Americas, by Clevenger *et al.* (2016).

The primary goal of this study was to develop global gene expression atlas showing genome‐wide expression of transcripts/genes in different tissues during the course of entire plant lifecycle of subsp. *fastigiata* of cultivated groundnut. This study sampled 20 tissues/organs throughout the life cycle of the cultivated groundnut (subsp. *fastigiata*) genotype ICGV 91114, a widely adapted high‐yielding Indian variety. The comprehensive RNA‐seq data analysis together with the reference genome for subsp. *fastigiata* provided the comprehensive gene expression atlas (AhGEA) for groundnut subsp. *fastigiata.* The in‐depth analysis of AhGEA provides insights on seed development, gravitropism and photomorphogenesis associated gene expression pattern, expression of different allergens during various stages of seed development and oil biosynthesis. The AhGEA for subsp. *fastigiata* developed in this study together with transcriptome map for subsp. *hypogaea* (Clevenger *et al.*, 2016) should be very useful in various functional, comparative and translational genomics studies in groundnut.

## Results

### Genome‐wide transcript expression profile

**Figure 1:**
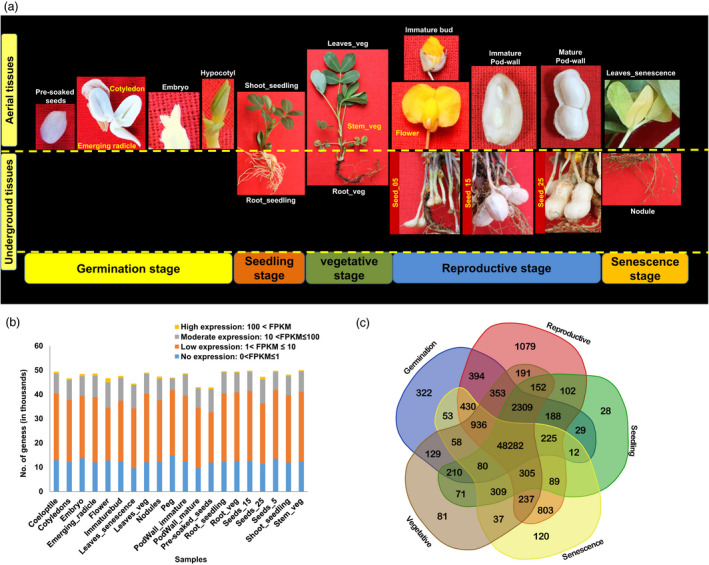
Samples used for developing groundnut gene expression atlas (AhGEA). (a) The 20 tissues targeted five growth stages including germinal (cotyledons, emerging radicle, hypocotyl, embryo and pre‐soaked seeds), seedling (root_seedling, and shoot_seedling), vegetative (leaves_veg, root_veg and stem_veg), reproductive (immature bud, flower, peg, seeds_05, seeds_15, seeds_25, pod wall_immature and pod wall_mature) and senescence (nodules and leaves_senescence). (b) Proportion of transcripts expressed at different levels (based on FPKM) in 20 tissues during the five plant growth stages. The bars indicated the number of transcripts expressed in each sample. Transcripts were categorized based on their expression: (i) no expression (0 < FPKM ≤ 1), (ii) low expression (1 < FPKM ≤ 10), (iii) moderate expression (10 < FPKM ≤ 100) and high expression transcripts (100 < FPKM). (c) Venn diagram of the numbers of shared and specifically expressed transcripts in germinal, seedling, vegetative, reproductive and senescence stage tissues.

The RNA‐sequencing approach was used to profile the transcriptome of 20 tissues including 13 aerial and seven underground tissues from five different plant growth stages (germinal, seedling, vegetative, reproductive and senescence). The stages include germinal (cotyledons, emerging radicle, hypocotyl, embryo, and pre‐soaked seeds), seedling (root_seedling, and shoot_seedling), vegetative (leaves_veg, root_veg, and stem_veg), reproductive (immature bud, flower, peg, seeds_05, seeds_15, seeds_25, pod wall_immature, and pod wall_mature) and senescence (nodules and leaves_senescence) (Figure 1a).

A total of 535.35 million raw reads were generated, and after the quality filtration, 480.31 million paired‐end filtered reads (89.72% of the total reads) were used for downstream analyses. On average, 95.84% of the high‐quality reads were mapped onto the reference genome for subsp. *fastigiata* of cultivated groundnut (Zhuang *et al.*, 2019) ranging from 81.60% in nodules to 98.10% in immature bud (Table S1). A gene was considered to be expressed if FPKM ≥ 1 (fragments per kilobase of exon per million reads mapped) in at least one sample and quantification status as ‘OK’. A list of expressed transcripts across the selected 20 tissues is given in Table S2. We identified expression of 57 344 transcripts in the 20 tissues ranging from 42 901 (Pre‐soaked seeds) to 49 983 (stem_veg). While categorizing the transcripts based on their expression, the largest portion of transcripts showed expression varying between 1 < FPKM ≤ 10 (55.1%, low expression), followed by moderate expression (ranged between 10 < FPKM ≤ 100, 17.7%) and highly expressed transcripts (100 < FPKM, 1.5%). It was noted that 25.8% of transcripts showed expression 0 < FPKM ≤ 1 (considered as no expression for this study) (Figure 1b). The proportion of transcripts expression in the above‐mentioned four expression categories were similar across stages.

**Figure 2:**
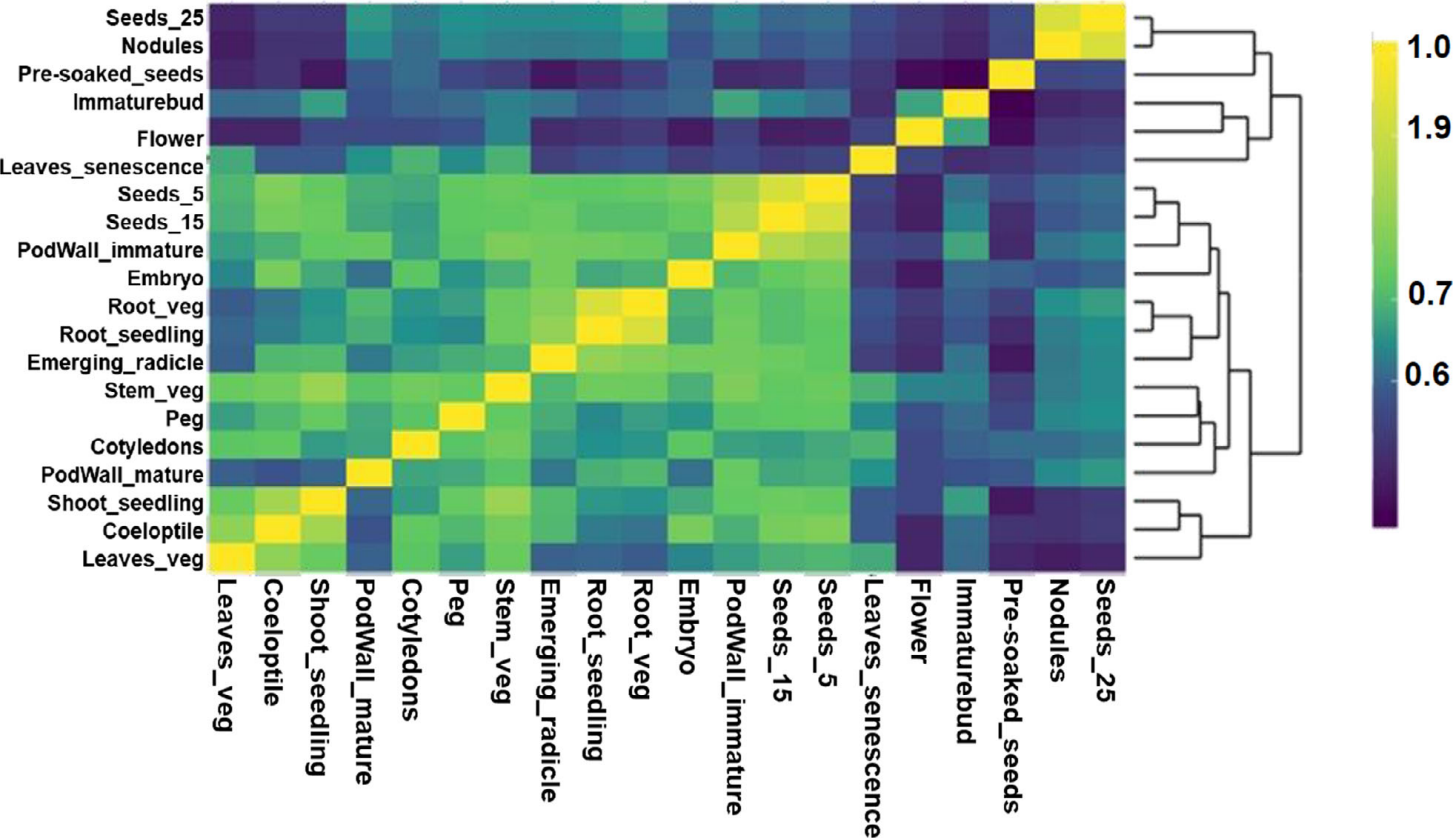
Grouping of tissues according to the global transcript expression patterns. Heatmap of Pearson's correlation‐based hierarchical clustering (using corrplot and heatmaply function in R) of 20 tissues selected for developing the AhGEA. Genes with a normalized expression level (FPKM ≥ 1) in at least one of the 20 tissues analysed were log2‐transformed and are designated as expressed. The colour scale indicates the degree of correlation. Samples were clustered based on their pairwise correlations.

Comparison among the transcripts from the five developmental stages of groundnut showed that the number of transcripts specific to a development stage was smallest in seedling stage (28), while reproductive stage had the largest expressed transcripts number (1079). There was a high degree of overlap in the number of transcripts expressed in different tissues and shared between the five developmental stages. A set of 48 282 transcripts were shared among the five groundnut developmental stages and could be referred to as the ‘core’ expressed transcripts (Figure 1c). We used the entire gene expression profiles of the 20 tissues/ organs to examine their relatedness of gene expression patterns. The biological identity of tissues from the five different plant growth stages reflected well in the respective transcriptome expression as revealed by hierarchical clustering using Pearson's correlations (Figure 2). The distribution of expressed transcripts based on their functional annotation revealed a higher representation of the metabolic and cellular process, catalytic and binding activity, membrane, cell and cell part (Figure S1). There are several transcripts present in plants involved in basic biological processes. These transcripts are expected to express across tissues (constitutive expression) and believed to perform ‘housekeeping’ functions. In order to identify the constitutively expressed transcripts in the selected 20 tissues, we calculated the coefficient of variation (CV) of each transcript. Using a threshold of CV ≤ 10%, a total of 467 constitutively expressed transcripts are identified in the present analysis. Further assessment of the selected constitutively expressed transcripts showed a very low standard deviation in expression values ranging from 0.17 to 0.80. (Table S3). Analysis of the identified constitutively expressed transcripts revealed their involvement in basic biological processes like cell division, protein and ion transport, regulation of transcription, and DNA repair.

We also analysed the transcripts that were specifically expressed in one tissue by considering a cut‐off of tissues specificity index ‘1’ (see Methods). High expression of such transcripts was detected in pre‐soaked seeds (157), followed by flower (153). The lowest number of specific transcripts was observed in pod wall_immature (8), coleoptile, podwall_immature and shoot_seedling (10; Table S4). As expected, it was observed that transcripts encoding for photosynthetic electron transport chain, cellulose biosynthetic process and cell wall organization were active in peg, which may be involved to initiate seed formation process. Similarly, the expression of phosphorylation and protein phosphorylation was also observed to be elevated which is associated with senescence by modulating several metabolic activities. A heatmap was constructed using the log_2_‐transformed FPKM values to visualize these transcripts across all different tissues indicating their tissue specificity (Figure S2 and Table S4).

### Expression patterns of transcription factor (TF) genes

Transcription factors are the key regulators, which mediate the transcriptional regulation. Among the studied 20 groundnut tissues, 26 187 transcripts encoded for TF were identified which could be categorized into 61 TF families. It was noted that the bHLH was the most abundant TF family followed by NAC, MYB‐related, ERF, C2H2 and WRKY (Figure S3a). These TF families are well characterized as an important regulatory component in transcriptional networks, controlling diverse developmental processes. Groundnut has a specialized organ called peg (Kumar *et al.*, 2019). While analysing the TF families in the peg, we found that to response to darkness and mechanical stimulus, transcripts encoding for GRAS, MYB‐related, bHLH and B3 TF families were expressed which in turn influence expression of hormone signalling genes for peg gravitropic response. Similarly, TFs are known to play a major role in flower development. Therefore, we looked into the transcripts encoding for TFs in flower development. In total, nine and 21 TFs were present in immature_bud and flower, respectively, of that all the nine TFs present in immature_bud were common in flower tissue as well. Among the various TF families, MYB, MYB‐related, NAC and bHLH encoding transcripts were the most represented in immature_bud and flower tissues. Further, to get insight into tissue‐specific TFs, we looked into the TF families known to play important regulatory functions in nodulation. We found that transcripts encoding for bHLH, ARF, ERF, NAC, FAR1 and NF‐Y TF families were expressed in the nodules reflecting their role in groundnut nodule formation (Figure S3b).

### Gene expression pattern during seed development and nodulation

**Figure 3:**
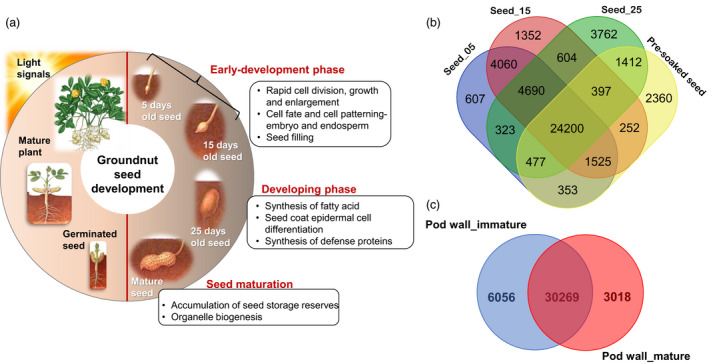
Seed development in groundnut. (a) A glimpse of the important biological processes involved during various stages of groundnut seed development. (b) Venn diagram of transcripts identified across four seed samples. (c) Venn diagram of transcripts identified in immature and mature pod walls. Venn diagrams were created using http://bioinfogp.cnb.csic.es/tools/venny/.

Seed development is a very important trait that determines yield, hence becomes imperative to understand the molecular mechanisms underpinning seed development (Figure 3a). In this regard, the transcripts expressed in six samples related to seed development were studied. Principal component analysis (PCA) was conducted to transform these sample variables into subsets that would provide information on analysing the samples for meaningful biological interpretation (He *et al.*, 2008). PCA performed on six samples, namely seed_05, seed_15, seed_25, pre‐soaked seeds, pod wall_immature and pod wall_mature. The subset‐I included seed_05, seed_15, and pod wall_immature which are in the early seed development stage, while subset‐II (seed_25) represented the developing stage whereas subset‐III constituted of pre‐soaked seed and pod wall_mature samples (Figure S4). Thus, the sample variables could be categorized as subsets representing the early development (subset‐I), developing (subset‐II) and the mature (subset‐III) stages of groundnut seed development (Figure S4).

Based on the reads mapped to the recently published groundnut allotetraploid genome belonging to subsp. *fastigiata* (Zhuang *et al.*, 2019), a total of 48 181 transcripts were expressed in all the six samples. A total of 8081 transcripts were exclusively expressed in seed_05 (607), seed_15 (1352), seed_25 (3762) and pre‐soaked seeds (2360) transcripts, while 24 200 transcripts were shared among the selected four seed samples (Figure 3b). Similarly, 30 269 transcripts were found to be common among immature and mature pod wall samples, whereas 6056 (pod wall_immature) and 3018 (pod wall_mature) transcripts were exclusively expressed in the two samples (Figure 3c). These transcripts were further studied based on their specific expression for their role in various biological processes and metabolic functions involved during groundnut developmental process.

During the early seed developmental stage represented by subset‐I, 4060 transcripts were exclusively shared among seed_05 and seed_15, while 607 and 1352 transcripts were specifically expressed, respectively (Figure 3b). The transcripts common to both the samples are mainly involved in rapid division, expansion and differentiation with a developmental program for cell fate, cell patterning and orientation for normal embryo and endosperm development (Table S5). Synthesis and assembly of photosystem/ cytochrome complex and cell wall modification are other predominant activities. Seed filling and seed composition are determined by activities including bidirectional sugar transporter (SWEETs), GDSL esterase/lipase and stearoyl‐[acyl‐carrier‐protein] 9‐desaturase six involved in carbohydrate and lipid metabolism, including ABC transporters involved in the translocation of fatty acids in the peroxisomes. Auxin‐mediated regulation is clearly indicated during this phase with highly expressed transcripts encoding auxin efflux carrier component, auxin response factor, auxin transporters, auxin‐binding protein, auxin‐induced protein and auxin‐responsive proteins. Several ethylene‐responsive transcription factors, MADS‐box proteins and MYB‐like proteins were expressed during this stage.

Seed_25 (subset‐II) representing the developing phase exclusively expressed 3762 transcripts that seem to have major role in fatty acid biosynthesis, metabolism and accumulation of oil bodies which determines the seed composition. Enzymes such as GDSL esterase/lipases, phospholipase‐like, were expressed during this stage. Calcium signalling and regulation is quite evident during this stage with the identification of transcripts encoding several calcineurin‐like protein, calcium ion‐binding protein, calcium/calmodulin‐regulated receptor‐like kinase 1 and calcium‐transporting ATPase. Defence‐related transcripts were also synthesized such as BON1‐associated protein 2, disease resistance protein, stilbene synthase 3 protein and pathogenesis‐related proteins, in addition to several MADS‐box protein, Zinc finger protein, bHLH, MYB and WRKY transcription factors. The complete list of transcripts expressed in seed samples of subset‐II has been provided in Table S5. Subset‐III comprised of pre‐soaked seed sample representing seed maturation stage and identified 2360 specifically expressed transcripts (Table S5). The most abundant proteins in subset‐III include late embryogenesis abundant protein, pentatricopeptide repeat‐containing protein, seed maturation protein, seed storage protein classes Ara h1.

To get insight into groundnut nodulation, we identified a total of 35 201 transcripts in the root nodules (Table S6). These nodule‐specific transcripts included those encoding for wound‐induced protein, cell cycle checkpoint control protein RAD9A, cell differentiation protein RCD1 homolog, cell division control protein 2 homolog C, cell division cycle 5‐like protein isoform X1, cell division protein FtsY homolog, cell division topological specificity factor homolog, and cell number regulator 5‐like. Other transcripts included early nodulin protein, nodule inception (NIN), nodulation receptor kinase‐like, nodulin 21 ‐like transporter family, nodulation‐signalling pathway, glutamine synthetase nodule isozyme and homeobox protein having important role in nodule organogenesis.

### Deciphering gravitational signals and photomorphogenesis in groundnut

**Figure 4:**
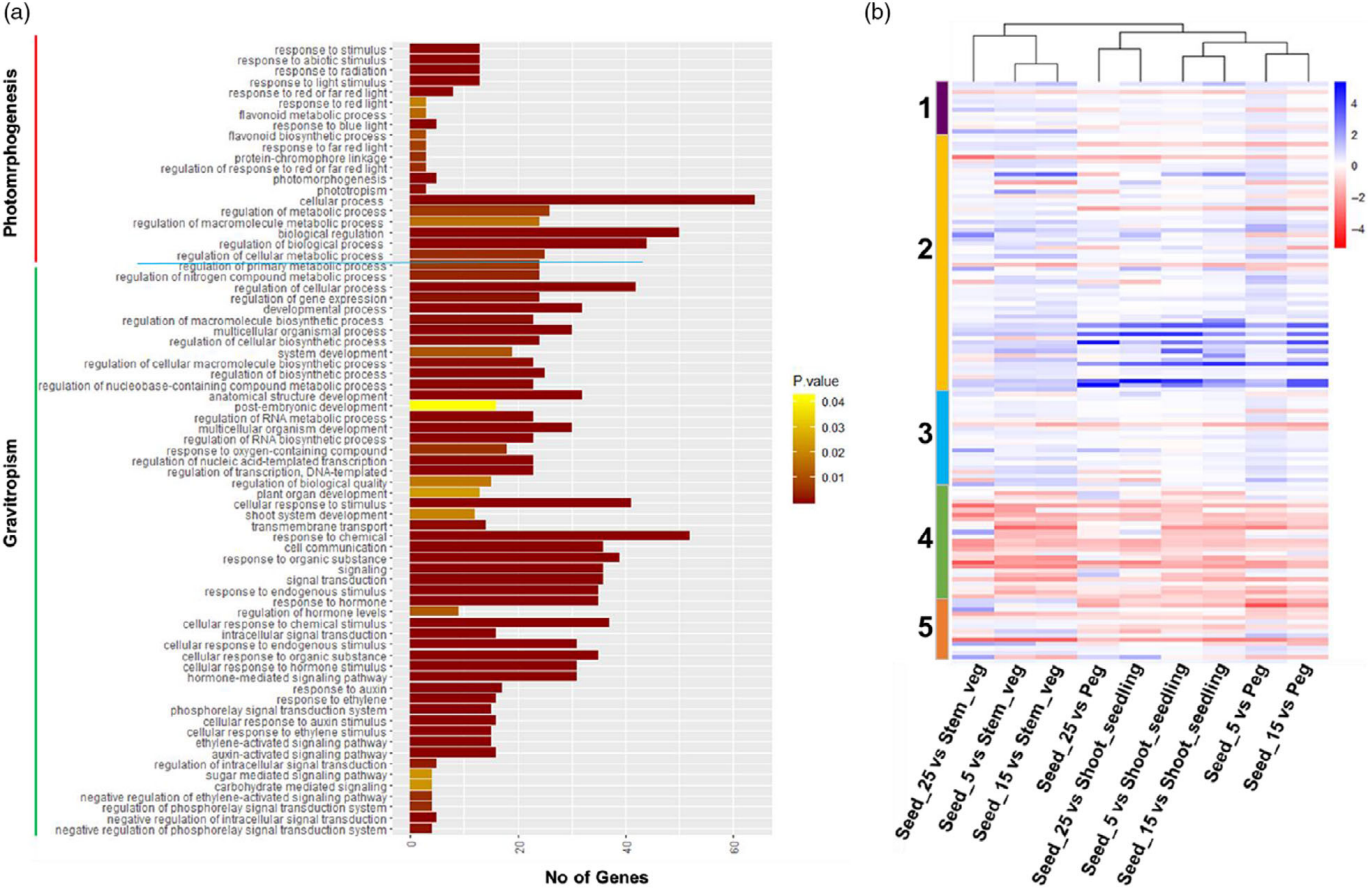
Expressed transcripts associated with gravitropism and photomorphogenesis. (a) Gene ontology analysis depicting the number of transcripts enriched for gravitropism and photomorphogenesis with their corresponding functional annotation. The colour of the horizontal axis shows the enrichment P‐values, the maximum of 0.05 in yellow and minimum in red. (b) The heatmap represents the expression (log transformed FPKM values) of differentially expressed genes (DEGs) involved in (1) embryonic development (purple bar), (2) gravitropism (orange bar), (3) hormone synthesis and response (blue bar), (4) photomorphogenesis (green bar), and (5) photosynthesis and light signal transduction (red bar). The DEGs have been identified from the nine pairwise comparison, namely seed_5 vs peg, seed_15 vs peg, seed_25 vs peg, seed_5 vs shoot_seedling, seed_15 vs shoot_seedling, seed_25 vs shoot_seedling, seed_5 vs stem_veg, seed_15 vs stem_veg, seed_25 vs stem_veg. First three categories mentioned above (related to gravitropism) have up‐regulated genes, and the other two categories (related to phototropism) have down‐regulated genes collectively. The blue color represents up‐regulation (FPKM ≥ 1) and red color denotes downregulation (FPKM ≤ 1) of the transcripts.

A comprehensive analysis of differentially expressed transcripts was performed to decipher the molecular mechanisms involved in gravitational signals (gravitropism) and photomorphogenesis. We took six tissues from which three were from above the ground (shoot_seedling, stem_veg, peg) and three from below the ground (seeds_5, seeds_15 and seeds_25), from three different seed developmental stages. Based on GO enrichment analysis, we identified 135 transcripts expressed associated with photomorphogenesis (26 transcripts), embryonic development (13 transcripts), gravitropism (59 transcripts), hormone biosynthesis and response (21 transcripts) and photosynthesis and light signal transduction (15 transcripts; Table S7, Figure 4a).

The combinatorial comparison of 135 transcripts selected under different categories between subterranean and overhead tissues provided us with the deferentially expressed genes with a flow of explaining the gravitropism and photomorphogenesis gene expression pattern. The DEGs were denoted in the heatmap showing the expression patterns of seeds_5, seeds_15 and seeds_25 against peg, shoot_seedling and stem_veg in their respective combinations (Figure 4b). It was observed that genes categorized under hormone synthesis and response, gravitropism and embryonic development are up‐regulated, whereas genes categorized under photomorphogenesis and photosynthesis and light signal transduction are down‐regulated in seed tissues. Beginning from peg to seeds_5 stage, the gravitropism‐related genes were much regulated in seeds_5 tissue than seeds_15 and seeds_25. It explains the signals of gravity received from peg stops, and the embryonic development‐related genes start expressing after it introduces itself in subterranean region. It was noted that after getting into a dark area, the hormone synthesis and response‐related genes such as auxin, ethylene and brassinosteroid pathways start expressing changes in its behaviour as a root. Gibberellin‐related genes were expressed higher in seeds_25 tissue than seeds_5 and seeds_5 indicating its property of cell senescence and seizing of response to gravity. The genes related to photomorphogenesis and photosynthesis and light signal transduction were reduced in all the three seed tissues which explain the absence of light. The 135 transcripts were enriched with GO terms with *P*‐value ≤0.05 (Figure 4a) by using Panther GO version 14. More than 40 transcripts were concomitant with response to stimulus, response to chemical, regulation of cellular process, regulation of biological process, cellular response to stimulus, cellular process and biological regulation. Our study revealed the exact path of seed development beginning from a circle of attaining a sense of gravity to losing in pod development.

### Global expression of allergen encoding transcripts

**Figure 5:**
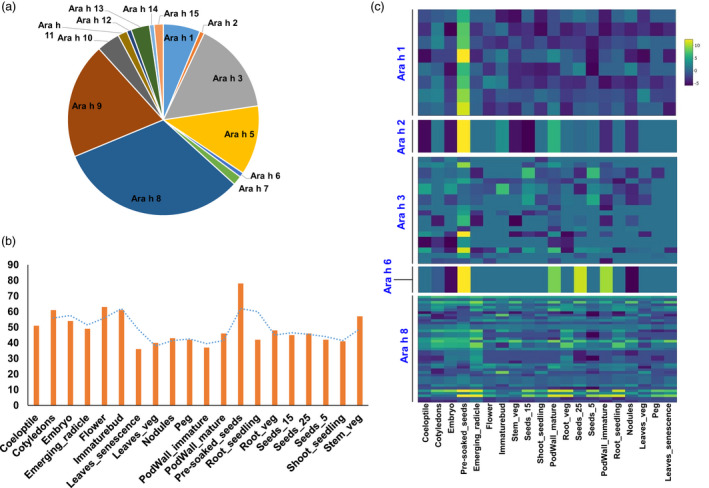
Global expression of allergen encoding transcripts across 20 selected groundnut tissues. (a) Distribution of allergens encoding transcripts across 20 selected groundnut tissues, (b) number of expressed transcripts across the tissues, (c) expression patterns of four major (Ara h1, Ara h2, Ara h3 and Ara h6) and one minor (Ara h8) across the selected 20 tissues.

To gain insights into the groundnut allergen encoding transcripts, we searched for the allergens encoding transcripts at the whole transcript level in the studied 20 tissues. Analysis of the selected 20 tissues of groundnut for allergenic proteins identified 14 isoallergens and variants encoded by 128 transcripts (Table S8). During the analysis, we found that the most abundant allergens identified were Ara h 8 (41 transcripts) followed by Ara h 9 (25 transcripts), Ara h 3 (20 transcripts) and Ara h 5 (15 transcripts; Figure S5). It was noted that Ara h 15 encoding transcripts were found in almost all the tissues (90%) followed by Ara h 5 (59.33%) and Ara h 8 (58.05%; Figure 5a). Among the different types of allergens identified so far, Ara h 1, Ara h 2, Ara h 3, Ara h 6 and Ara h 8 are the most frequently recognized allergens (Ratnaparkhe *et al.*, 2014). All these frequently recognized allergens were present in most of the groundnut tissues selected for our analysis, and it ranged from 28.13% (leaves_senescence with 36 allergens encoding transcripts) to 60.94% (pre‐soaked seed with 78 allergens encoding transcripts) (Table S9). However, in terms of allergens, Ara h 8 transcripts were found expressed across all the 20 studied tissues.

Although Ara h 15 and Ara h 5, to date, are not reported as the major allergens, our data suggested that an extended detailed examination is required to get in‐depth information because both of the allergens were expressed across most of the selected tissues. In agreement with earlier reports that allergens mainly act as the maturity approaches, we observed that pre‐soaked seeds (stored seed soaked in water for 24 h) contained transcripts (60.94% of total 128 expressed transcripts) encoding for all types of isoallergens except Ara h 2 and Ara h 12. (Figure 5b). Among the various isolated allergen types, four allergens (Ara h 1 with 8 transcripts and Ara h 3 with 20 transcripts members of the cupin superfamily, Ara h 2 with 1 transcript and Ara h 6 with 1 transcript members of the prolamin superfamily) are major and one (Ara h 8 with 41 transcripts; member of the pathogenesis‐related protein 10 (PR‐10) class of proteins (Riecken *et al.*, 2008) is minor allergen related to the food allergy and majorly expressed in pre‐soaked seeds (Figure 5c).

### Triacylglycerol and fatty acid synthesis‐related genes

**Figure 6:**
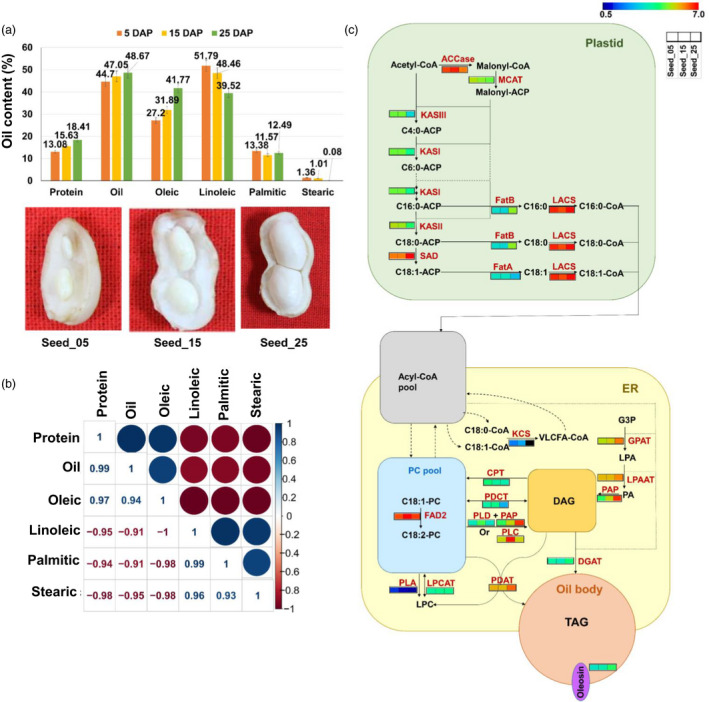
Oil content and transcript expression pattern in developing groundnut seeds. (a) Phenotyping of oil and four different fatty acids at three different seed developmental stages, (b) Pairwise correlation of oil, protein and four different fatty acids for three seed developmental stages, (c) A conceptual diagram of transcripts encoding for enzymes related to fatty acid biosynthesis and TAG synthesis pathways.

The total oil content along with the major fatty acid (FA) composition of seeds derived from three developmental stages namely 5 days after peg enters the soil 5DAP (seed_05, early seed development stage), 15DAP (seed_15, mid seed development stage) and 25DAP (seed_25, late seed development stage) of ICGV 91114 was measured. As shown in Figure 6a, oil starts to accumulate at the early stage of seed development (seed_05, 44. 70%) and after late stage (seed_25, 48.67%), majority of oil gets accumulated in the seeds. The phenotypic variability of oleic acid gradually increased from early to late seed maturity stages, ranged from 27.20% (seed_05) to 41.77% (seed_25). Contrastingly, percentage of linoleic acid, palmitic acid and stearic acid gradually decreased from early to late seed development stages and range was from 39.52% (seed_25) to 51.79% (seed_05), 11.57% (seed_25) to 13.38% (seed_05) and 0.08% (seed_25) to 1.36% (seed_05), respectively. As expected, during all the stages of oil accumulation, stearic acid was in the lowest proportion. Correlation analysis clearly displayed a significant and a negative correlation of oleic acid with linoleic acid (*r* = −0.996, *P* < 0.0001), stearic acid (*r* = −0.980, *P* < 0.001), and palmitic acid (*r* = −0.981, *P* < 0.0001). A significant positive correlation was observed for linoleic with palmitic acid (*r* = 0.987, *P* < 0.0001), stearic acid (*r* = 0.926, *P* < 0.001) and palmitic acid with stearic acid (*r* = 0.962, *P* < 0.01; Figure 6b).

Considering the significance of groundnut as an oil crop, functional annotation and GO annotation, transcriptome profiling of developing seeds identified 877 transcripts coding for oil biosynthesis in groundnut (Table S10). Investigation of transcripts encoding biological pathways involved in oil biosynthesis in groundnut identified 74 transcripts associated with fatty acid (FA) synthesis, four transcripts associated with FA elongation, 14 transcripts associated with Kennedy pathway, 46 transcripts associated with acyl‐CoA independent pathway, and six transcripts for oil body (oleosin; Tables S11 and S12). Results suggested that the five (glycerolipid metabolism, glycerophospholipid metabolism, FA degradation, FA biosynthesis and FA elongation) oil biosynthesis‐related biological pathways were activated for triacylglycerol (TAG) assembly during seed development process. The schematic metabolic pathways leading to oil accumulation in groundnut have been integrated and compiled in Figure 6c.

Fatty acid synthesis is localized into plastids. As shown in Figure 6c, expression of transcripts for MCAT encoding for Malonyl CoA‐acyl carrier protein transacylase, KASI, KASII and KASIII encoding for Ketoacyl‐ACP Synthase I, II and III, respectively, and SAD encoding for Stearoyl‐ACP Desaturase which are found in the group of plastid fatty acid synthesis has a significantly elevated expression level in the early and medium stages of seed development (seed_05 and seed_15). After FA synthesis in plastid, these acids were transported to the endoplasmic reticulum (ER). Similarly, in the early and medium stages of seed development, transcripts for KCS encoding for Ketoacyl‐CoA Synthase, FAD2 encoding for Oleate Desaturase, PLA encoding for Phospholipase A2, PLC encoding for Phospholipase C, PLD encoding for Phospholipase D & delta and PDCT encoding for phosphatidylcholine:diacylglycerol choline phosphotransferase showed higher expression level in comparison with late seed_25. In both acyl‐CoA‐independent and the acyl‐CoA‐dependent pathways, all the TAG biosynthesis‐related transcripts encoding for enzymes that catalysed the two pathways were found to be significantly expressed.

Transcripts encoding for the key genes involved in the acyl‐CoA‐independent pathway with high level of expression were observed for diacylglycerol choline phosphotransferase (CPT), phosphatide cytidylyltransferase (PAP), 1‐acylglycerol‐3‐phosphocholine acyltransferase (LPCAT) and phospholipid: diacylglycerol acyltransferase (PDAT) during medium and late stages of seed development (seed_15 and seed_25). In the case of acyl‐CoA‐dependent pathway known as the Kennedy pathway, transcripts for GPAT encoding for glycerol‐3‐phosphate acyltransferase, LPAAT encoding for 1‐acylglycerol‐3‐phosphate acyltransferase, PAP encoding for phosphatide cytidylyltransferase, GPAT encoding for glycerol‐3‐phosphate acyltransferase and DGAT encoding for acyl‐CoA: diacylglycerol acyltransferase have significantly higher expression at medium and later stages of seed development. Interestingly, it was observed that there were transcripts which have similar expression pattern across different seed developmental stages such as ACCase encoding for acetyl‐CoA carboxylase, LACS encoding for long chain acyl‐CoA synthetase and FAD2 encoding for omega‐6 fatty acid desaturase. At the end of the TAG synthesis pathways, there was an elevated expression of transcript encoding for the structural protein, oleosin during the later stage of seed development (seed_25), which is the main structure‐stabilizing protein of the plant oil bodies. In our study, six transcripts were identified encoding for oleosin.

## Discussion

In contrast to most animals, plant development is a continuous process where new organs keep on initiating and elaborating throughout the life cycle. This reflects that many transcriptional regulations underlying the development of different organ system are continuously active in plants. In order to develop a genome‐wide gene expression profile for plant parts involving key tissues and growth stages, this study reports and made available a global gene expression atlas for subsp. *fastigiata* of cultivated tetraploid groundnut. This atlas will serve as the basic reference for expressed genes in groundnut and will facilitate improved understanding of the biological processes underlying growth and development for a wealth of features and phenomena in groundnut.

### AhGEA: an essential resource for functional genomics research in groundnut

Global gene expression profiling data generated in the present study covering different tissues of different developmental stages will be useful towards the understanding gene functions in the biological processes. The developed data could be utilized in functional genomics research to pin‐point targeted candidate genes in groundnut. Trait mapping efforts led to identification of associated genomic regions for several important traits in groundnut including biotic and abiotic stresses, agronomic, yield component and oil quality traits (Agarwal *et al.*, 2018; Khedikar *et al.*, 2018; Pandey *et al.*, 2012, 2016, 2017; Varshney *et al.*, 2013; Vishwakarma *et al.*, 2017). Most of these studies identified mere loosely associated markers. AhGEA developed in this study together with earlier available resources will accelerate discovery of causal genes and understanding the molecular mechanism of the targeted traits. Of the two widely grown subspecies (*hypogaea* and *fastigiata)* across the world, the varieties from subsp. *fastigiata* cover maximum area, mostly in Asia and Africa, while subsp. *hypogaea* being more in Americas. The gene expression atlas has already been developed earlier (Clevenger *et al.*, 2016) for subsp. *hypogaea* while this study now makes available atlas for highest cultivated subsp. *fastigiata* for global research community. Most importantly, this study has used the reference genome of same subsp. (*fastigiata* genome by Zhuang *et al.*, 2019) for entire analysis indicating a high‐quality and reliable gene expression atlas for use in different functional and translational genomics research in groundnut.

Transcripts displaying tissue‐specific gene expression are important in understanding the specialized processes occurring within these tissues. In our study, we identified transcripts with specific tissue expression (specific to only one tissue) which represents significant information for targeted gene expression. For instance, over‐representation of transcripts encoding for predictable specialized functional classes related to inorganic phosphate transporter and calcium‐transporting ATPase endoplasmic reticulum‐type was expressed in pod wall. Similarly specialized transcripts encoding for seed coat and oil bodies were identified in seeds. This study also provides the stable transcripts with the least variability among tissues involved in basic metabolic processes. It is confirmed from many previous studies that it is very crucial to use reference genes specific to the trait under study and various plant developmental stages (Reddy *et al.*, 2013; dos Santos *et al.*, 2018; Sinha *et al.*, 2015). Such information on tissue‐specific as well as constitutively expressed genes is not available for groundnut research community. Therefore, availability of such information through this study on highly stably expressed genes may be useful as reference for future gene expression analysis of developmental studies in groundnut.

### Insights into seed biology and nodulation

Groundnut seed development is one of the most important processes from an agronomic point of view. From the crops perspective, it is a storehouse of energy and nutrients for use in future for the developing embryos and the seedling. Therefore, seeds undergo development through a highly regulated and ordered series of events underground. During this period, food reserves in the form of oil, protein and carbohydrate are formed and accumulated, which serves as a major source of nutrition for human and animal consumption. Here, we decipher the transcriptomic response during different stages of groundnut seed development, from five DAP to seed maturity. It was found that the seed_25 has been predominantly associated with accumulation of storage protein (legumin type B, arachin Ahy‐3‐like) and seed oil (seipin‐1 isoform X2, oleosin, fatty acid desaturase). Primarily, the fructification is followed by accumulation of storage compounds such as proteins, oils and fats through various biological processes and molecular functions during seed development.

Groundnut has an excellent ability to fix symbiotic nitrogen fixation compared to most of the tropical legume. Unlike the common mode of rhizobial infection through the root hair in most legumes, groundnut seems to get infected by means of crack entry at the lateral root emergence with intercellular spreading to form determinate nodules (Tajima *et al.*, 2008). The intercellular penetration of the bacterium could lead to a preferential expression of disease resistance, defensin‐like and pathogenesis‐related proteins. Following nodule infection, there is a prolific nodule organogenesis possibly in cortical cells leading to a mature active nodule. Non‐legume haemoglobin‐like proteins are highly synthesized in the nodule which provide oxygen to the rhizobium for symbiotic nitrogen fixation. Nodule transcriptome in the present study also showed the expression of transcripts encoding CLAVATA3/ESR (CLE)‐related protein, CLE protein implying an auto‐regulation of nodulation.

### Elucidating genes involved in gravitropism and photomorphogenesis

As an embryo is the start of life, phototropism and gravitropism are the start of life cycles in plants. Two tropisms have been discussed over two centuries on how it influences in the biological regulation in greens to survive independently. Light and dark reactions have always been negatively co‐regulated, and its expression is inversely proportional. Breaking down the queries of plant development by light intensities in every aspects of anatomy, interest is also shown in one rare category called geocarpy. The plants develop its fruits beneath the soil are categorized under geocarpy. Sensibly, the groundnut is defined as geophytic geocarpy as they are supposed to be an aerial flowering by processing with burial of ovary below the ground covering with an organ of shell known as peg or gynophore (Barker, 2005). The transformation of peg to seed undergoes four types of biological regulation such as (i) gravity sensing or losing phototropism, (ii) auxin signalling, (iii) ethylene and gibberellin signalling, and (iv) embryonic development (Chen *et al.*, 2016b; Kumar *et al.*, 2019; Li *et al.*, 2017). By defining the gravitropic‐related genes, they have been categorized in two such as positive gravitropism and negative gravitropism. The peg moves towards the soil by the sense of gravity. In particularly concentrating on the Phototropism and gravitropism, after the fertilization, the peg is formed by pausing the growth of embryo and carries the ovary at its tip (Band *et al.*, 2012). The gravity is sensed by the statocyst of starch‐filled amyloplasts in columella cells, which is accumulated in the lateral elongation zone. Even though the statocysts responses to the gravity, polarized auxin efflux carrier components (PIN) direct the peg into the soil for a linear development. Auxin efflux carriers (PIN‐formed) are distributed asymmetrically in the peg which leads to bury itself into the ground (Krecek *et al.*, 2009). PINOID (PID) also plays an important role in enhancing auxin transport in association with PIN proteins for organ development. The growth of embryo is resumed by the combined action gibberellin and kinetin in producing starchless amyloplasts, which results in the loss of gravitropism resulting in no longer need to intrude into the soil. By untying the knots of our results obtained and connecting it to the above references, we discuss here it as transcripts of genes regulating phototropism gradually decreased in all the seed tissues when compared with peg by exposing it into the darker area and loses its help from light. *AUXIN EFFLUX CARRIER COMPONENT (PIN)* along with *PINOID (PID)* expresses higher in seed_15 and seed_25 stages to stimulate the auxin polar transport mechanism between the cells. *PINOID* binds with calcium binding proteins such as *TOUCH3 (TCH3)*, a calmodulin‐related protein, and *PID‐BINDING PROTEIN 1 (PBP1)*, which is expressed higher in the presence of auxin, mediates the level of calcium proves its participation in auxin‐mediated signalling pathway for plant growth development (Benjamins *et al.*, 2003). As peg enters into ground, hormonal pathways start regulating such as ethylene and gibberellin signalling in the seed tissues as ethylene is produced more in the darkness and gibberellin mediates the growth development. As the hormones come to play in plant development, the embryonic‐related genes also got expressed higher in the seeds, resulting in a smooth embryo development. The important note is the observation of the transcripts obtained between the tissues. The hormone biosynthesis and embryonic development happen only after seed_15 in the scope of plant development explaining the expectation of increased auxin level. Gibberellin‐related genes are expressed highly at the seed tissue seed_5 and seed_15, explaining its contribution to the plant development at the seed_5 stage and its contribution to the loss of gravitropic response. At seed_15 stage, it was noted that the cytokinin levels are higher indicating that combined work of gibberellin and kinetin‐related genes seizing the sense of gravity. Our results clearly suggest that the term of gravitropic response starts from peg to the seed at 15 days.

### Better insights on expression dynamism of allergens in different plant parts

Groundnut allergens can trigger a potent and sometimes dangerous immune response in an increasing number of people (Mueller *et al.*, 2014). Groundnut seed contain over 32 different proteins but only 18 have been reported to have an allergen property and 17 (Ara h 1 to Ara h 17) have been identified so far (http://www.allergen.org). Among the various isolated allergens from groundnut, four are major (Ara h 1 and Ara h 3; members of the cupin superfamily of proteins, Ara h 2 and Ara h 6; members of the prolamin superfamily) and one minor allergen (Ara h 8; member of the pathogenesis‐related protein 10 (PR‐10) class of proteins) regard to food allergy (Iqbal *et al.*, 2016; Ratnaparkhe *et al.*, 2014; Zhou *et al.*, 2013). Currently, the only effective treatment for groundnut allergy is avoidance of the food due to lack of global understanding of allergens associated transcripts in different tissues, non‐availability of cost effective and rapid screening protocols resulting into non‐availability of allergens free genotypes. Recent studies from our group reported development of ELISA‐based protocol for estimation of allergens from groundnut seeds (Pandey *et al.*, 2019b) and also identified several hypoallergen groundnut genotypes (Pandey *et al.*, 2019c). Our findings address multiple questions regarding the identification of allergens encoding transcripts and their expression variation in different tissues. We have identified large number (128) of allergens encoding transcripts for twelve different allergens and their expression in 20 different tissues. As expected, the allergens encoding transcripts were more expressed in seed tissues and consistent with earlier reports that allergens belong to storage protein family, and in our study, we observed highest level of expression in pre‐soaked seeds. The transcript expression of four major allergens (Ara h 1, Ara h 2, Ara h 3 and Ara h 6) was high in seed tissues. Surprisingly, the transcript expression of Ara h 8, reported to be a minor allergen was found across all the tissues. Collectively, through generating a highly resolved and extensive transcriptome map of groundnut, a solid framework for a systemic approach was set to understand allergens associated genes and their expression across the tissues. This information could be utilized in better understanding of allergens associated genes and together with development of rapid screening protocols and availability of lines with low level of allergens. Combined information from our and several other studies could be useful in developing allergen‐free lines.

### Oil biosynthesis progressively increased till seed maturity

The three seed development stages (seed_05, seed_15 and seed_25) were selected for oil biosynthesis analysis when rapid increase in storage product synthesis and the seed biosynthetic pathways are found at their maximum activity. Analysis identified approximately 2800 transcripts encoding for enzymes associated with metabolic pathways involved in glycerophospholipid metabolism, glycerolipid metabolism and fatty acid metabolism. Global transcriptome data of the seed samples were analysed for identifying the genes associated with oil formation, concentration or composition. This work provides a complicated picture of the enzymatic steps leading to the biosynthesis of TAG. In this study, we analysed all of the seed oil‐related genes. Global expression data of potential lipid metabolic pathways including the classical Kennedy pathway (GPAT, LPAAT, PAP, DGAT), acyl‐CoA‐independent pathway (CPT, PDCT, PAP, PLC, PDAT) and FA biosynthesis (ACCase, KAS, SAD, FatA, FatB, LACS) were used to develop a more comprehensive and precise biosynthetic pathway for TAG biosynthesis in groundnut seeds (Yin *et al.*, 2013; Yu *et al.*, 2015). The temporal expression patterns of some important genes involved in oil biosynthesis showed that the oil bodies' stability‐related oleosin was expressed strongly at seed_25; in contrast, other TAG and FA synthesis‐related genes were expressed at relatively higher level at seed_05 and seed_15 samples As indicated by Yu *et al.* (2015) in their study, our results also indicated that seed first accumulates high levels of the enzymes for oil biosynthesis in the early stage of seed development as compared to the late stage (seed_25). Contrastingly, the biosynthetic rate of proteins (oleosin) for the oil bodies' stability was at high point in the latter stage (seed_25) when the accumulation of seed oil was high. Thus, the oleosin genes were strongly expressed to provide enough proteins to sustain the oil bodies stabilization and prevent them from coalescence during their sustained and rapid growth.

Phenotyping of three important seed developmental stages involved in oil accumulation in groundnut for oil content and its different components revealed a gradual increase for oleic acid and decrease for linoleic, palmitic and stearic acids from early to late seed developmental stages. Similar observation was also reported in groundnut where seed development stages showed a similar pattern of gradual increase and decrease of different fatty acid compounds (Yin *et al.*, 2013). Also, an inverse correlation was observed for oleic acid vs linoleic acid, stearic acid and palmitic acid (Pandey *et al.*, 2014; Shasidhar *et al.*, 2017). The association between different fatty acid composition has recently been deployed through introgression of mutant FAD alleles to develop high oleic groundnut lines for health benefits to consumers and extended shelf life of groundnut products (Janila *et al.*, 2016). In conclusion to best of our knowledge, this is one of the most complete and extensive for TAG biosynthesis‐related genes in groundnut. The datasets developed in this study together with previously published information's will enhance the genomic resource database for groundnut. These resources will be useful for better understanding of oil accumulation pathways and to help in genomics‐assisted breeding (GAB) for development of high oleic lines.

## Conclusion

In the present study, we performed transcriptome profiling of *Arachis hypogaea* for the world's widest cultivated subsp. *fastigiata* using 20 diverse tissues covering entire developmental stages. We identified 57 344 transcripts significantly expressed across all the major plant tissues/ organs. Out of these expressed transcripts, we identified 88.79% annotated genes mapped onto the reference genome for subsp. *fastigiata* of cultivated groundnut. All the generated resources have been deposited to NCBI SRA database and also have been provided as supplementary Tables. These resources will be very useful for accelerating functional and translational genomics research in groundnut. For examples, by using this gene expression atlas, we have provided a baseline description of the molecular mechanisms occurring during complex regulatory networks such as gravitropism and photomorphogenesis, seed development, allergens and oil biosynthesis in groundnut. Additionally, we have identified a set of stable expressing transcripts across the selected tissues, which could be used as an internal control in groundnut functional genomics studies.

## Materials and methods

### Plant materials

In the present study, *Arachis hypogaea* L. cultivar ‘ICGV 91114’ belonging to Spanish botanical variety of subsp. *fastigiata* of cultivated tetraploid groundnut was used to develop gene expression atlas. This cultivar is a widely adapted variety in many states of India and is popular due to being high‐yielding, drought‐tolerant and early‐maturing variety. Seeds were grown in three independent biological replicates for different set of plants to ensure reproducibility of the results. A total of 20 tissues covering entire life cycle of groundnut plant, including 13 aerial and seven underground, were collected from five plant growth stages namely, germinal (5), seedling (2), vegetative (3), reproductive (8) and senescence (2).

In brief, seeds were surface sterilized for 10 min with chloride solution (6 g/L) and germinated in Petri dishes at room temperature for harvesting tissues from the germinal stage. Another set of seeds were sown under glasshouse conditions, and tissues were harvested from the seedlings after 7 days of germination. Further, the seedlings were allowed to grow in plastic pots filled with autoclaved black soil, sand and vermicompost (10 : 10 : 1 v/v) mixture under controlled glasshouse conditions. Fresh tissues from different organs such as leaves, stems and roots were harvested from all three growth stages, namely vegetative, reproductive and senescence, respectively, and frozen immediately in liquid nitrogen till RNA extraction. Similarly, nodules at senescence, immature bud flowers and peg at reproductive stage were harvested and tissues sampled independently for RNA isolation. For the seed developmental and oil biosynthesis studies, when each gynophore penetrated the soil surface and was buried in the soil (pegging) date was noted, and each plant was labelled. At a specific developmental stage of 5, 15 and 25 days after pegging (DAP), designated as seed_05, seed_15 and seed_25 respectively, seeds were collected and frozen in liquid nitrogen for RNA extraction. The immature and mature pod walls were collected from seed_05 and seed_25, respectively. A concise description of the tissues collected to generate global gene expression atlas is given in Table S1 and presented in Figure 1.

### RNA extraction and sequencing

Three independent biological replicates of the selected 20 tissues/organs were used for constructing mRNA sequencing libraries. Each library was sequenced in a single lane as described previously by Pazhamala *et al.* (2017) and Kudapa *et al.* (2018). Total RNA was isolated from all the 20 selected tissues using RNeasy Plant Mini Kit (Qiagen, Hilden, Germany). The quality and quantity of each RNA sample were assayed using NanoDrop spectrophotometer (Thermo Scientific, Wilmington, DE) and the Agilent 2100 Bioanalyzer (Agilent Technologies, Santa Clara, CA). Subsequently, 20 libraries were prepared for whole transcriptome sequencing using an Illumina TruSeq RNA Sequencing Kit following manufacturer's instructions (Illumina, San Diego, CA). The mRNA libraries were then quantified using a Qubit 2.0 fluorometer (Thermo Fisher Scientific, Waltham, MA) and validated using an Agilent 2100 Bioanalyzer. Further, cluster generation for these libraries was done on cBot, followed by 100bp paired‐end sequencing on HiSeq 2500 platform (Illumina).

### Data processing

The raw data for each sample were checked for quality using FastQC v0.11.6 (https://www.bioinformatics.babraham.ac.uk/projects/fastqc/). Low‐quality reads (<Q20) and adapter sequences were filtered through Trimmomatic v0.32 (Bolger *et al.*, 2014). Post‐trimming, the data were analysed using the Tuxedo pipeline. The filtered reads were aligned to cultivated groundnut (*Arachis hypogaea*) reference genome using Tophat2 v2.1.1 and Bowtie2 v2.3.5 (Trapnell *et al.*, 2012). The alignment bam files from Tophat2 for each sample along with reference genome GFF was used to perform RABT (reference annotation‐based transcript) assembly through Cufflinks v2.2.1. The obtained cufflink assemblies were then compared and merged using cuffmerge script from cufflinks to remove transfrags and generate a combined GTF for further downstream analysis. Further, cuffdiff from Cufflinks was used to estimate the abundance in terms of fragments per kilobase of exon per million reads mapped (FPKM) and to identify differentially expressed genes (DEGs). A transcript was considered to be expressed when FPKM ≥ 1 in at least one sample, and a gene was said to be significantly differentially expressed when |log_2_(fold change)| ≥ 2 with and *P*‐value ≤ 0.05. The identified transcripts were then annotated against NCBI's non‐redundant (nr) protein database (taxon. *viridiplantae*) using standalone blast‐2.7.1+ (e‐value ≤ 10^−5^) followed by Gene Ontology (GO) and KEGG pathway analysis using Blast2GO v5.2.

### Identification of constitutively and tissue‐specific expressed transcripts

Constitutively expressed transcripts were identified using coefficient of variation (CV), where CV was calculated as ratio between standard deviation (*σ*) and mean (*µ*) for log2(FPKM + 1) values for each transcript across the samples. The stably expressed genes were identified according to their coefficients of variation (CV ≤ 10%) and their stability measurements.

The tissue‐specific transcripts were identified by calculating tissue specificity index (τ) (Yanai *et al.*, 2005) using the equation:

$$\tau =\frac{\sum_{i-1}^{N}\left(1-x_{i}\right)}{N-1},$$

where *N* is the number of samples and *x_i_* is the expression value of a gene normalized by maximum value across all samples. The value of *τ* ranges from 0 to 1, where higher the value more likely the gene is specifically expressed in that stage. For this study, genes with *τ* = 1 were considered as tissue specific.

### Transcript analysis of groundnut‐specific traits

Groundnut trait‐specific analysis for gravitropism and photomorphogenesis, seed development, allergens and oil biosynthesis was performed based on annotation prediction. Log‐transformed values were used for projection of all the expressed data across tissues. For Gene ontology (GO) enrichment analysis associated with gravitropism and photomorphogenesis, genes from groundnut were matched and extracted with the arabidopsis genes in The Arabidopsis Information Resource, version 10 (TAIR10) (www.arabidopsis.org/aboutarabidopsis.html) on www.arabidopsis.org, and performed GO enrichment analysis using Panther v.14.0 (Mi *et al.*, 2019).

## Conflict of interest

The author(s) declare that they have no competing interests.

## Author contributions

RKV conceived the idea and supervised the study. PS performed most of the analysis. PS, PB and LTP interpreted the results and wrote the manuscript. AC and AD contributed to data generation. PS, PB and AWK carried out bioinformatic analysis. MKP, SN, DH, HJ, WZ, BG and BL contributed to analysis and interpretation of results. All authors read and approved the final manuscript.

## Supporting information


**Figure S1** Gene Ontology annotation of expressed transcripts.
**Figure S2** Heatmap of tissue‐specific expressed transcripts in groundnut.
**Figure S3** Abundance distribution of transcription factor families in the 20 selected groundnut tissues.
**Figure S4** Principal component analysis (PCA) of six seed and pod wall samples.
**Figure S5** Isoallergens and variants encoding transcripts expressed across selected 20 groundnut tissues.
**Table S1** Summary of RNA‐sequencing reads mapped to reference assembly.
**Table S2** List of expressed transcripts (FPKM>1) across selected 20 groundnut tissues.
**Table S3** A list of the most stably expressed transcripts.
**Table S4** A list of tissue‐specific expressed genes.
**Table S5** Specifically expressed transcripts of seed sample from subset‐I, subset‐II and subset‐III.
**Table S6** Transcripts exclusively expressed in nodules.
**Table S7** Expressed transcripts related to gravitropism and photomorphogenesis.
**Table S8** A list of isoallergens and variants identified in the selected 20 groundnut tissues.
**Table S9** A list of allergen encoding transcripts across the 20 selected tissues.
**Table S10** A list of expressed transcripts coding for oil biosynthesis in groundnut.
**Table S11** List of key enzymes identified in the present study related to TAG synthesis and FA metabolism in groundnut.
**Table S12** Groundnut transcripts in TAG biosynthesis pathways.

## Data Availability

All sequencing data generated have been deposited into National Center for Biotechnology Information (NCBI) Sequence Read Archive (SRA) database under the BioProject ID: PRJNA484860.
