# Supplementary material for: Arachis hypogaea gene expression atlas for fastigiata subspecies of cultivated groundnut to accelerate functional and translational genomics applications
Source: Plant Biotechnol J. 2020 Apr 23;18(11):2187–200. doi: 10.1111/pbi.13374 (PMC7589347; doi:10.1111/pbi.13374)
Supplement: Supplementary file 1 — Figure S1 Gene Ontology annotation of expressed transcripts. Figure S2 Heatmap of tissue‐specific expressed transcripts in groundnut. Figure S3 Abundance distribution of transcription factor families in the 20 selected groundnut tissues. Figure S4 Principal component analysis (PCA) of six seed and pod wall samples. Figure S5 Isoallergens and variants encoding transcripts expressed across selected 20 groundnut tissues. Table S1 Summary of RNA‐sequencing reads mapped to reference assembly. Table S2 List of expressed transcripts (FPKM>1) across selected 20 groundnut tissues. Table S3 A list of the most stably expressed transcripts. Table S4 A list of tissue‐specific expressed genes. Table S5 Specifically expressed transcripts of seed sample from subset‐I, subset‐II and subset‐III. Table S6 Transcripts exclusively expressed in nodules. Table S7 Expressed transcripts related to gravitropism and photomorphogenesis. Table S8 A list of isoallergens and variants identified in the selected 20 groundnut tissues. Table S9 A list of allergen encoding transcripts across the 20 selected tissues. Table S10 A list of expressed transcripts coding for oil biosynthesis in groundnut. Table S11 List of key enzymes identified in the present study related to TAG synthesis and FA metabolism in groundnut. Table S12 Groundnut transcripts in TAG biosynthesis pathways. [file PBI-18-2187-s001.zip › pbi13374-sup-0001-Supinfo/pbi13374-sup-0001-Supinfo.docx]

**Supplementary Information**

**The gene expression atlas for *fastigiata* subspecies of cultivated groundnut to accelerate functional and translational genomics applications**

Pallavi Sinha^1, †^, Prasad Bajaj^1, †^, Lekha T Pazhamala^1^, Spurthi N Nayak^1, 2^, Manish K Pandey^1^, Annapurna Chitikineni^1^, Dongxin Huai^3^, Aamir W Khan^1^, Aarthi Desai^1^, Huifang Jiang^3^, Weijian Zhuang^4^, Baozhu Guo^5^, Boshou Liao^3^, Rajeev K Varshney^1,^*

^1^Center of Excellence in Genomics & Systems Biology (CEGSB), International Crops Research Institute for the Semi-Arid Tropics (ICRISAT), Hyderabad, India

^2^Department of Biotechnology, University of Agricultural Sciences (UAS), Dharwad, India

^3^Oil Crop Research Institute (OCRI), Chinese Academy of Agricultural Science (CAAS), Wuhan, China

^4^College of Plant Protection, Fujian Agriculture and Forestry University (FAFU), Fuzhou, China

^5^USDA-ARS Crop Protection and Management Research Unit (CPMRU), Tifton, USA

^†^Authors contributed equally

**^*^Author for Correspondence**

Rajeev K Varshney

Center of Excellence in Genomics & Systems Biology

International Crops Research Institute for the Semi-Arid Tropics (ICRISAT)

Patancheru - 502 324, India

Telephone: 91-40-30713305;

Fax: 91-40-30713074

E-mail: [r.k.varshney@cgiar.org](mailto:r.k.varshney@cgiar.org)

**Table S1.** Summary of RNA-sequencing reads mapped to reference assembly

| Sample | Stage | Total raw reads (millions) | Total filtered  Reads (millions) | Mapped reads (%) | Transcripts expressed |
| --- | --- | --- | --- | --- | --- |
| Cotyledons | Germinal | 22.17 | 19.86 | 97.40 | 46628 |
| Emerging radicle | Germinal | 24.66 | 23.03 | 97.10 | 48598 |
| Coeloptile | Germinal | 33.01 | 30.68 | 98.00 | 49354 |
| Embryo | Germinal | 28.41 | 26.46 | 98.00 | 48432 |
| Root_seedling | Seedling | 29.43 | 27.50 | 97.60 | 49542 |
| Shoot_seedling | Seedling | 22.26 | 20.35 | 97.70 | 48148 |
| Immature bud | Reproductive | 25.37 | 23.30 | 98.10 | 47617 |
| Flower | Reproductive | 29.81 | 27.64 | 96.70 | 46699 |
| Seeds_05 (5 DAP) | Reproductive | 36.05 | 31.55 | 97.80 | 49737 |
| Seeds_15 (15 DAP) | Reproductive | 34.19 | 30.12 | 97.50 | 49795 |
| Pre-soaked seeds | Germinal | 25.77 | 22.76 | 97.50 | 42901 |
| Leaves_veg | Vegetative | 32.67 | 30.21 | 97.10 | 48978 |
| Root_veg | Vegetative | 31.67 | 28.80 | 96.40 | 49424 |
| Stem_veg | Vegetative | 22.85 | 20.80 | 96.60 | 49983 |
| Peg | Reproductive | 25.27 | 22.05 | 98.00 | 46910 |
| Seeds _25 (25 DAP) | Reproductive | 24.16 | 22.58 | 81.70 | 47253 |
| Nodules | Senescence | 27.21 | 17.95 | 81.60 | 47369 |
| Pod wall_immature | Reproductive | 32.08 | 29.41 | 97.50 | 48715 |
| Pod wall_mature | Reproductive | 10.00 | 9.67 | 97.30 | 42960 |
| Leaves_senescence | Senescence | 18.31 | 15.58 | 97.10 | 44452 |

DAP: Days after peg enters into the soil

**Table S2** List of expressed transcripts (FPKM>1) across selected 20 groundnut tissues.

**Detailed Excel file has been attached.**

**Table S3** A list of the most stably expressed transcripts.

**Detailed Excel file has been attached.**

**Table S4** A list of tissue-specific expressed genes.

**Detailed Excel file has been attached.**

**Table S5** Specifically expressed transcripts of seed sample from subset-I, subset-II and subset-III.

**Detailed Excel file has been attached.**

**Table S6** Transcripts exclusively expressed in nodules.

**Detailed Excel file has been attached.**

**Table S7** Expressed transcripts related to gravitropism and photomorphogenesis

**Detailed Excel file has been attached.**

**Table S8** A list of Isoallergens and variants identified in the selected 20 groundnut tissues.

**Detailed Excel file has been attached.**

**Table S9.** Percent expression of allergen encoding transcripts across the 20 selected tissues

| Isoallergens | Number of transcripts expressed (A) | Total number of tissues (B) | Max expected possibilities to express a transcript (A×B) across tissues | Number of transcript expressed across tissues | Percent (%) of expressed transcripts across tissues |
| --- | --- | --- | --- | --- | --- |
| Ara h 1 | 8 | 20 | 160 | 23 | 14.37 |
| Ara h 2 | 1 | 20 | 20 | 2 | 10 |
| Ara h 3 | 20 | 20 | 400 | 39 | 9.75 |
| Ara h 5 | 15 | 20 | 300 | 178 | 59.33 |
| Ara h 6 | 1 | 20 | 20 | 4 | 20 |
| Ara h 7 | 2 | 20 | 40 | 22 | 55 |
| Ara h 8 | 41 | 20 | 820 | 476 | 58.04 |
| Ara h 9 | 25 | 20 | 500 | 141 | 28.20 |
| Ara h 10 | 5 | 20 | 100 | 25 | 25.00 |
| Ara h 11 | 2 | 20 | 40 | 2 | 5.00 |
| Ara h 12 | 1 | 20 | 20 | 2 | 1.00 |
| Ara h 13 | 4 | 20 | 80 | 31 | 38.75 |
| Ara h 14 | 1 | 20 | 20 | 1 | 5.00 |
| Ara h 15 | 2 | 20 | 40 | 36 | 90 |

**Table S10** A list of expressed transcripts coding for oil biosynthesis in groundnut

**Detailed Excel file has been attached.**

**Table S11.** List of key enzymes identified in the present study related to TAG synthesis and FA metabolism in groundnut

| Pathway | Symbol | Enzyme (ko_definition) | Unigenes |
| --- | --- | --- | --- |
| Fatty Acid Synthesis | ACCase | Acetyl-CoA Carboxylase | 21 |
|  | KASI | Ketoacyl-ACP Synthase I | 2 |
|  | MCAT | Malonyl CoA-acyl carrier protein transacylase | 4 |
|  | KAS II | Ketoacyl-ACP Synthase II | 4 |
|  | KASIII | Ketoacyl-ACP Synthase III | 2 |
|  | FatA | Acyl-ACP Thioesterase A | 2 |
|  | FatB | Acyl-ACP Thioesterase B | 2 |
|  | SAD | Stearoyl-ACP Desaturase | 5 |
|  | LACS | Long-Chain Acyl-CoA Synthetase | 23 |
|  | FAD2 | Oleate Desaturase | 9 |
| Fatty Acid Elongation | KCS | Ketoacyl-CoA Synthase | 4 |
| Kennedy pathway | GPAT | Glycerol-3-Phosphate Acyltransferase | 14 |
|  | LPAAT | 1-Acylglycerol-3-Phosphate Acyltransferase | 11 |
|  | PAP | Phosphatidate Phosphatase | 7 |
|  | DGAT | Acyl-CoA : Diacylglycerol Acyltransferase | 6 |
| Acyl-CoA independent pathway | PDAT | Phospholipid : Diacylglycerol Acyltransferase | 12 |
|  | PDCT | Phosphatidylcholine:diacylglycerol cholinephosphotransferase | 3 |
|  | CPT | Diacylglycerol Cholinephosphotransferase | 7 |
|  | LPCAT | 1-Acylglycerol-3-Phosphocholine Acyltransferase | 2 |
|  | PLA | Phospholipase A2 | 3 |
|  | PLC | Phospholipase C | 14 |
|  | PLD | Phospholipase D &delta | 5 |
| Oil body biosynthesis | Oleosin | Oil-Body Oleosin | 6 |

**Table S12** Groundnut transcripts in TAG biosynthesis pathways

**Detailed Excel file has been attached.**

**
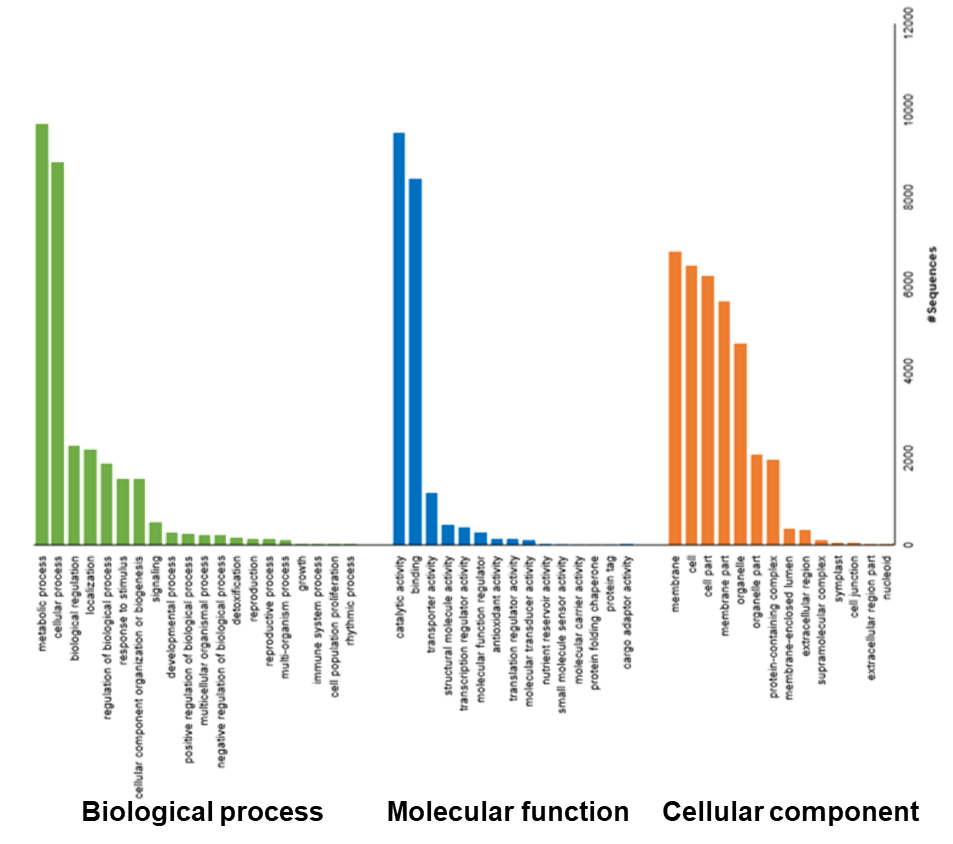
**

**Figure S1** Gene Ontology annotation of expressed transcripts. Bar graph representing GO annotations in three categories, (A) Biological Processes, (B) Molecular Function and (C) Cellular Component


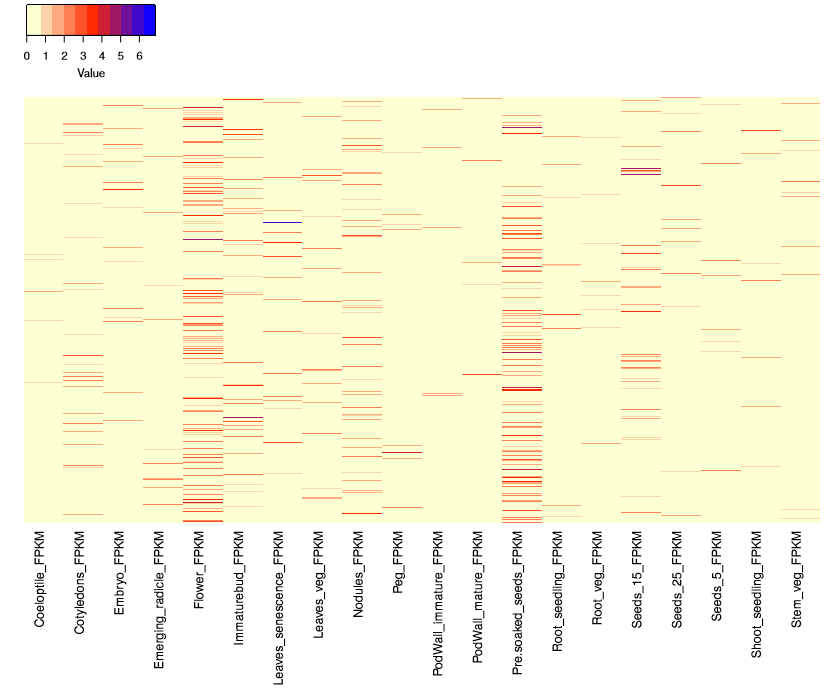


**Samples**

**Tissue specific expressed transcripts**


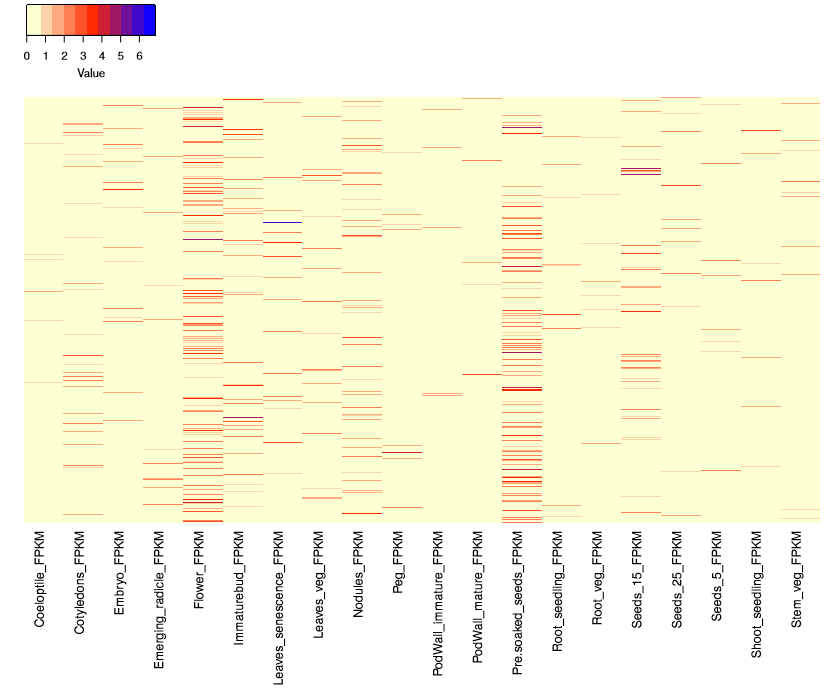


**Figure S2** Heatmap of tissue-speciﬁc expressed transcripts in groundnut. The color scale indicates the z-score associated with all the groundnut transcripts speciﬁcally expressed in a particular tissue/ organ.

**
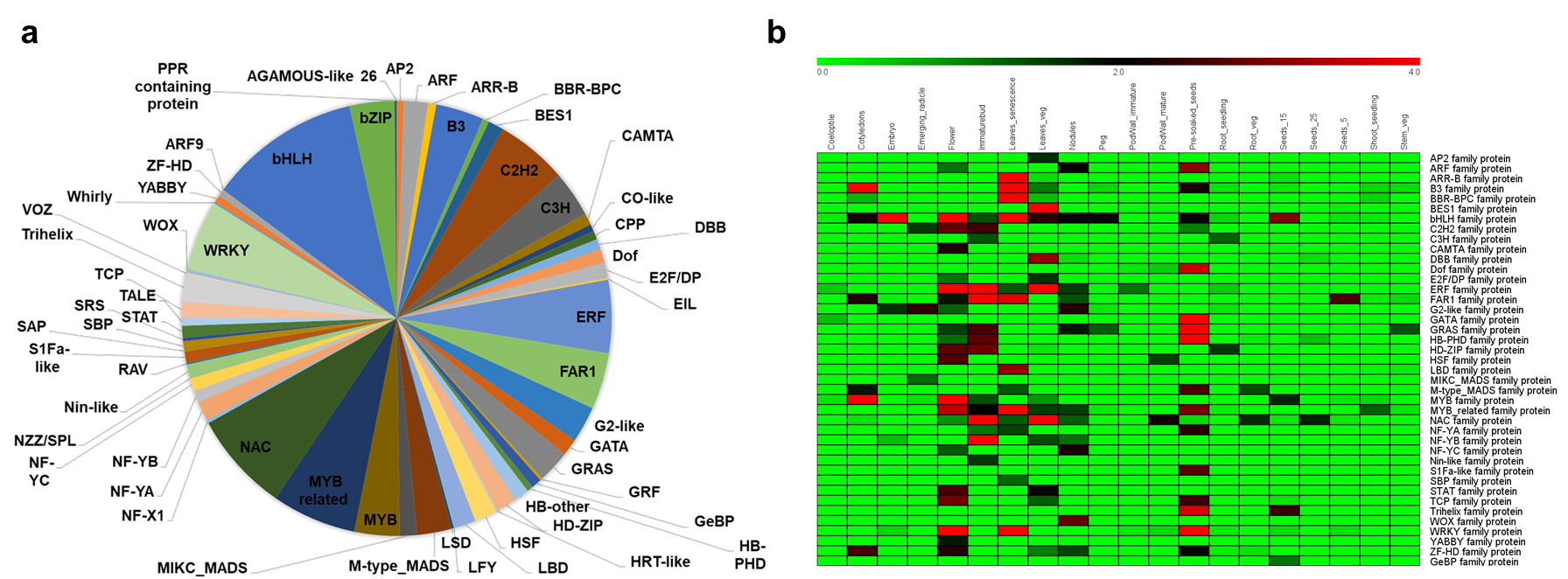
**

**Figure S3** Abundance distribution of transcription factor families in the 20 selected groundnut tissues. (a) Distribution of transcription factor encoding transcripts, based on their family membership. (b) Tissue specific transcription factors encoding transcripts across the 20 tissues.

**Figure S4** Principal Component analysis (PCA) of six seed and pod wall samples **
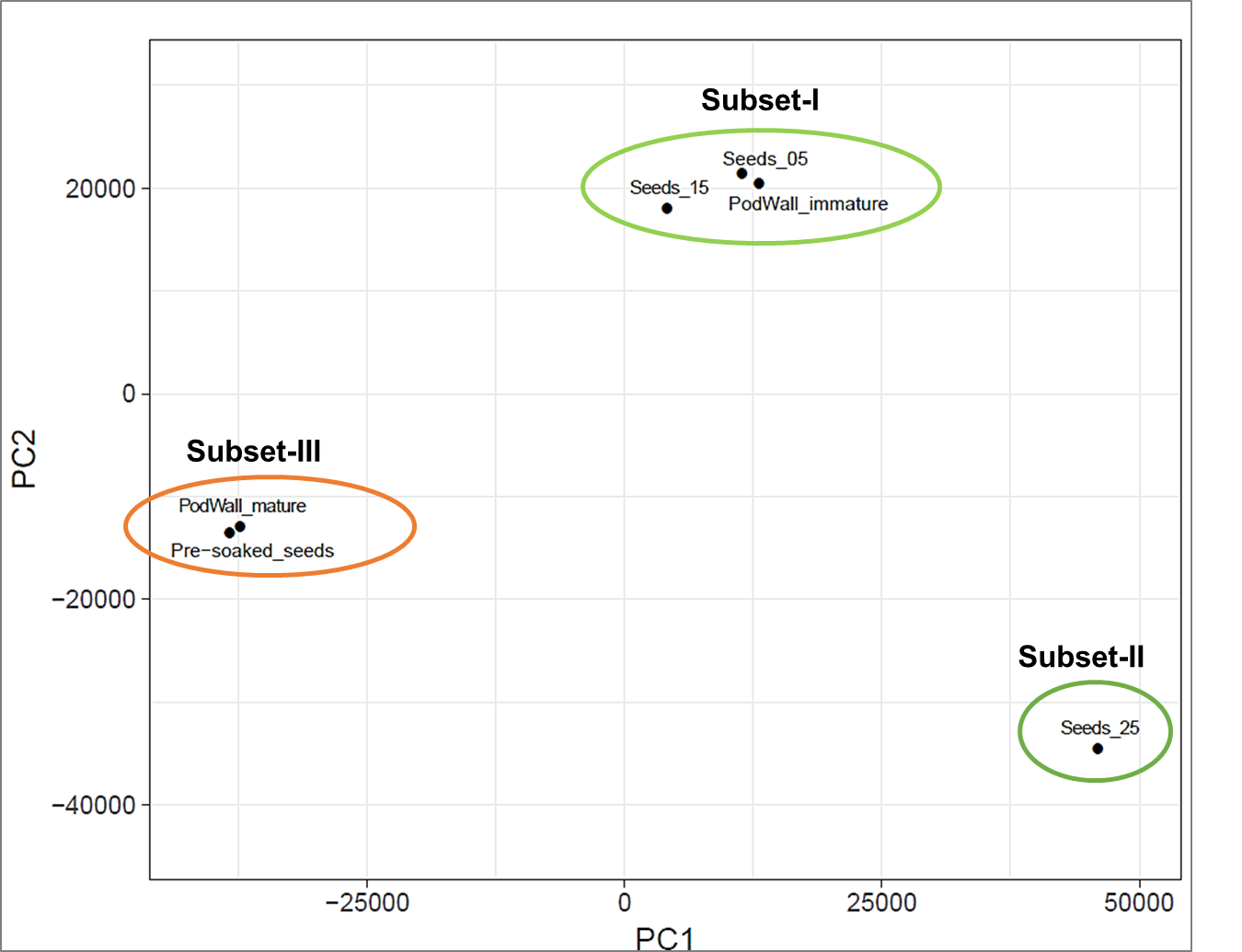
**

**
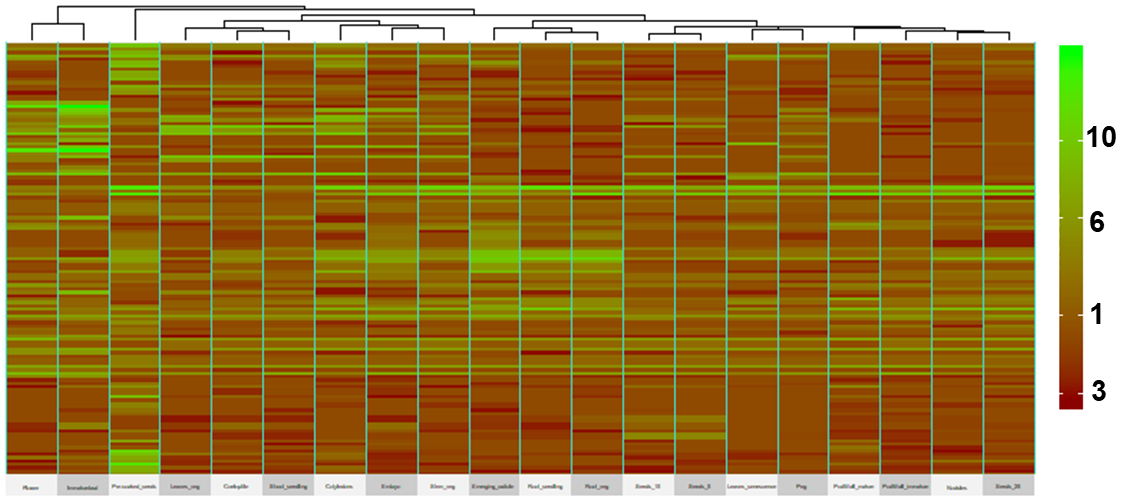
**

**Figure S5** Isoallergens and variants encoding transcripts expressed across selected 20 groundnut tissues
