# Supplementary material for: Arachis hypogaea gene expression atlas for fastigiata subspecies of cultivated groundnut to accelerate functional and translational genomics applications
Source: Plant Biotechnol J. 2020 Apr 23;18(11):2187–200. doi: 10.1111/pbi.13374 (PMC7589347; doi:10.1111/pbi.13374)
Supplement: Supplementary file 1 — Figure S1 Gene Ontology annotation of expressed transcripts. Figure S2 Heatmap of tissue‐specific expressed transcripts in groundnut. Figure S3 Abundance distribution of transcription factor families in the 20 selected groundnut tissues. Figure S4 Principal component analysis (PCA) of six seed and pod wall samples. Figure S5 Isoallergens and variants encoding transcripts expressed across selected 20 groundnut tissues. Table S1 Summary of RNA‐sequencing reads mapped to reference assembly. Table S2 List of expressed transcripts (FPKM>1) across selected 20 groundnut tissues. Table S3 A list of the most stably expressed transcripts. Table S4 A list of tissue‐specific expressed genes. Table S5 Specifically expressed transcripts of seed sample from subset‐I, subset‐II and subset‐III. Table S6 Transcripts exclusively expressed in nodules. Table S7 Expressed transcripts related to gravitropism and photomorphogenesis. Table S8 A list of isoallergens and variants identified in the selected 20 groundnut tissues. Table S9 A list of allergen encoding transcripts across the 20 selected tissues. Table S10 A list of expressed transcripts coding for oil biosynthesis in groundnut. Table S11 List of key enzymes identified in the present study related to TAG synthesis and FA metabolism in groundnut. Table S12 Groundnut transcripts in TAG biosynthesis pathways. [file PBI-18-2187-s001.zip › pbi13374-sup-0001-Supinfo/pbi13374-sup-0008-TableS9.docx]

**Table S9.** Allergen encoding transcripts across the 20 selected tissues

| Isoallergens | Number of transcripts expressed (A) | Total number of tissues (B) | Max expected possibilities to express a transcript (A×B) across tissues | Number of transcript expressed across tissues | Percent (%) of expressed transcripts across tissues |
| --- | --- | --- | --- | --- | --- |
| Ara h 1 | 8 | 20 | 160 | 23 | 14.37 |
| Ara h 2 | 1 | 20 | 20 | 2 | 10 |
| Ara h 3 | 20 | 20 | 400 | 39 | 9.75 |
| Ara h 5 | 15 | 20 | 300 | 178 | 59.33 |
| Ara h 6 | 1 | 20 | 20 | 4 | 20 |
| Ara h 7 | 2 | 20 | 40 | 22 | 55 |
| Ara h 8 | 41 | 20 | 820 | 476 | 58.04 |
| Ara h 9 | 25 | 20 | 500 | 141 | 28.20 |
| Ara h 10 | 5 | 20 | 100 | 25 | 25.00 |
| Ara h 11 | 2 | 20 | 40 | 2 | 5.00 |
| Ara h 12 | 1 | 20 | 20 | 2 | 1.00 |
| Ara h 13 | 4 | 20 | 80 | 31 | 38.75 |
| Ara h 14 | 1 | 20 | 20 | 1 | 5.00 |
| Ara h 15 | 2 | 20 | 40 | 36 | 90 |
